# Dynamic Clustering and Coordinated User Scheduling for Cooperative Interference Cancellation on Ultra-High Density Distributed Antenna Systems

**DOI:** 10.3390/e20080616

**Published:** 2018-08-19

**Authors:** Kazuki Maruta

**Affiliations:** Graduate School of Engineering, Chiba University, Chiba 263-8522, Japan; maruta@chiba-u.jp; Tel.: +82-43-290-3314

**Keywords:** distributed antenna, cell clustering, cooperation, large scale, multiuser MIMO

## Abstract

This paper proposes dynamic clustering and user scheduling for previously conceived inter-cluster interference cancellation scheme on ultra-high density distributed antenna system (UHD-DAS). UHD-DAS is composed of one central unit (CU) and densely deployed remote radio units (RUs) serving as small cell access points. It can enhance spatial spectral efficiency by alleviating traffic load imposed per radio unit; however, intenser small cell deployment revives the inter-cell interference (ICI) problem. Cell clustering, cooperation of multiple RUs, can mitigate ICI partially, whereas inter-cluster interference (ICLI) still limits its possible capacity. Simplified ICLI cancellation based on localized RU cooperation was previously proposed to mitigate interference globally. The resolved issue is that it required frequency reuse distance to fully obtain its interference cancellation ability. This paper introduces dynamic clustering with coordinated user scheduling to ensure reuse distance without extra frequency reuse. Joint dynamic clustering and ICLI cancellation can effectively work and almost reaches ideal performance as full cooperative spatial multiplexing transmission.

## 1. Introduction

Continuous evolution of mobile communication systems brings us versatile services engendering not only explosive data traffic but also the number of terminals. To accommodate the explosive increase in data traffic and terminals, small cell densification is the most straightforward means [1,2,3]. It contributes to load balancing since the number of user equipments (UEs) connected to base stations (BSs) can be lessened such that UEs can occupy spectral resources which are generally shared in time or frequency domains. Although this environment brings satisfactory signal-to-noise power ratio (SNR) even around the cell edge, inter-cell interference (ICI) becomes the dominant factor strongly limiting system capacity.

Meanwhile, enhancing transmission rate by spatial multiplexing known as multiple-input multiple-output (MIMO) [4] has been now standardized in many systems such as wireless fidelity (Wi-Fi), worldwide interoperability for microwave access (WiMAX) and long term evolution (LTE)(-Advanced). Multiuser MIMO (MU-MIMO) [5], in which the base station (BS) simultaneously communicates with multiple user equipments (UEs), can also effectively increase system capacity without sharing time/frequency spectral resources. The above concept can be expanded to multiple BSs regarding as distributed antenna elements, i.e., coordinated multipoint (CoMP) [6]. Basics of CoMP, that is, cooperative transmission [7,8], were widely investigated due to its effective spatial diversity and reduced energy consumption. Functionality of BS is assumed to be split into central unit (CU) and remote radio units (RUs) [9,10]. CU mainly incurs medium access control and base band signal processing for all RUs. RUs are connected to the CU with optical fibers and have radio frequency (RF) components serving as small cell access points. Since widely spread cooperating RUs offer low spatial correlation, this system architecture is well suited to MU-MIMO transmission. It organizes a virtual large macro cell where ICI is excluded and thus system capacity can be largely enhanced. With the single frequency reuse based cellular systems, cooperative transmission was expected to be an essential technique to improve the throughput performance for cell-edge users. This concept is now revived as ultra-high density distributed antenna systems (UHD-DAS) [11,12,13]. Here arises a challenge to realize large scale cooperation: excessive computation complexity imposed by multiuser beamforming/detection. Dividing all cooperative RUs into *clusters*, formed by a limited number of RUs, can alleviate such complexity. The transmission/reception weight can be determined cluster-by-cluster, and thus their matrix size becomes smaller. However, clustering causes co-channel inter-cluster interference (ICLI) again and cluster-edge users still suffer this problem. To address this issue, we previously proposed an ICLI cancellation scheme for large scale cooperation [14,15,16]. After the CU acquires all the ICLI signals and channel state information (CSI) including inter-cluster path, it generates the ICLI signal replica and subtracts from the original transmission signal. At the receiver side, ICLI components are cancelled out. The above signal processing can be conducted in a simple linear manner. Its overall computation complexity is not so significant compared to the conventional full cooperative MU-MIMO processing.

The ICLI cancellation so far necessitates the condition where the original desired signal strength must be larger than interference due to its simplified nature, otherwise the ICLI power is conversely emphasized. Our previous study was based on the strict frequency reuse [15,16] or fractional frequency reuse (FFR) [17,18] constraint to satisfy the above requirement in compensation for bandwidth division loss. To address this issue, this paper proposes to introduce dynamic clustering [19] and coordinated user scheduling which can leverage ICLI cancellation capability without any frequency reuse. System level simulations elucidate that the proposed scheme can achieve spectral efficiency performance approximately approaching the full cooperative MU-MIMO transmission.

The rest of the paper is organized as follows. Section 2 describes the system model of clustered UHD-DAS and ICLI cancellation scheme. Section 3 introduces dynamic clustering and coordinated user scheduling schemes. Computer simulation results are presented in Section 4. Finally, the paper is concluded in Section 5.

## 2. System Model and Schemes

**Figure 1:**
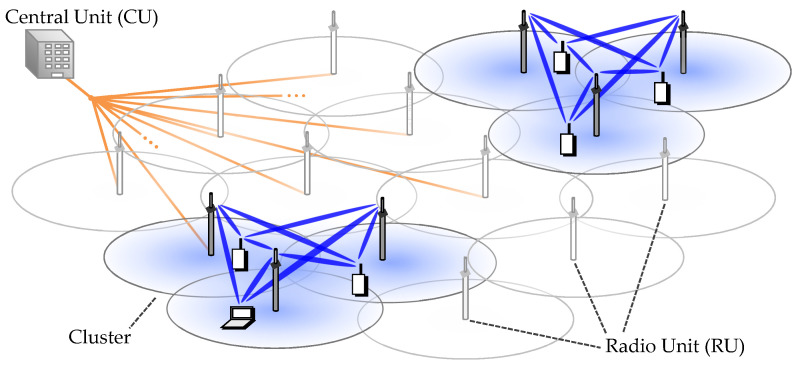
Cluster-based cooperative transmission in ultra-high density distributed antenna system.

In this paper, lowercase letters represent scalar quantities, hile bold letters indicate vectors or matrices. The system of interest assumes that *L* RUs are connected to one CU. The RUs in a cluster work cooperatively via MU-MIMO, except for the case that the cluster is composed of one cell. CU manages all medium access controls for RUs and calculates all transmission/reception weight matrices for MU-MIMO for clusters. The number of cooperating RUs are defined as the cluster size *C*. Figure 1 illustrates the cluster based cooperative transmission in UHD-DAS. In the figure, cluster size of three is exhibited as an example. Increasing cluster size *C* can weaken ICLI since the cooperating RUs are more widely isolated. On the contrary, small *C* is preferable from the viewpoint of the computation complexity required for MU-MIMO signal processing in respective clusters. Here, *L* RUs and scheduled *K* UEs (L=K) are assumed to communicate simultaneously via the same frequency channel. The full size downlink channel matrix $H\in C^{K\times L}$ is represented as,

(1) $$H=\left(\begin{array}{cccccc} h_{1,1} & h_{1,2} & & \ldots & & h_{1,L}\\ h_{2,1} & h_{2,2} & & & & \\ & & \ddots & & & \\ \vdots & & & h_{k,l} & & \vdots \\ & & & & \ddots & \\ h_{K,1} & & & \ldots & & h_{K,L} \end{array}\right),$$

where $h_{k,l}$ is the channel coefficient from the *l*-th RU to the *k*-th UE. When clustered channel matrices are introduced, **H** is then expressed as,

(2) $$H=\left(\begin{array}{cccccc} H_{1,1} & H_{1,2} & & \ldots & & H_{1,M}\\ H_{2,1} & H_{2,2} & & & & \\ & & \ddots & & & \\ \vdots & & & H_{i,j} & & \vdots \\ & & & & \ddots & \\ H_{M,1} & & & \ldots & & H_{M,M} \end{array}\right).$$

where $H_{i,j}\in C^{C\times C}$ denotes the clustered channel submatrices that include channel coefficients from RUs in the *j*-th cluster to UEs in the *i*-th cluster. Diagonal components (i=j) represent intra-cluster channel submatrices and non-diagonal components (i≠j) represent ICLI channel submatrices from the *j*-th cluster to the *i*-th cluster. *M* is the number of clusters, i.e., L=MC. The weight matrix $W\in C^{L\times K}$ is calculated by using arbitrary algorithms, but the computation complexity explosively increases as the size of H increases. Practical interference suppression methods for large scale cooperation (i.e., large size of H) with reasonable computation complexity were the target of this study.

### 2.1. Inter-Cluster Interference Cancellation

Let $W_{i}\in C^{C\times C}$ and $s_{i}\in C^{C\times 1}$ denote the precoding weight matrix for the *i*-th cluster generated from $H_{i,i}$ and the transmission symbols, respectively. the weighted initial transmission signal vector on the downlink at the *i*-th cluster $t_{i}\in C^{C\times 1}$ is expressed as,

(3) $$t_{i}^{\left(0\right)}=W_{i}s_{i},$$

Reception signal vector $r_{i}^{\left(0\right)}\in C^{C\times 1}$ for the UEs in the *i*-th cluster is then given by,

(4) $$\begin{array}{ccc} r_{i}^{\left(0\right)} & = & \sum_{j}^{M}H_{i,j}t_{j}^{\left(0\right)}\\ & = & H_{i,i}W_{i}s_{i}+\sum_{j\neq i}^{M}\left(H_{i,j}W_{j}s_{j}\right)+n_{i}, \end{array}$$

where $H_{i,i}W_{i}s_{i}\left(i=j\right)$ is the desired signal for UEs in the *i*-th cluster, $H_{i,j}W_{j}s_{j}\left(i\neq j\right)$ is the ICLI signal from adjacent *j*-th cluster, and $n_{i}$ denotes the additive white Gaussian noise (AWGN) vector. The proposed scheme subtracts interference signal replicas from initial $t_{i}^{\left(0\right)}$ by use of the knowledge of interference channel states and signals. The transmission signal vector with the first order ICLI cancellation is defined as,

(5) $$t_{i}^{\left(1\right)}:=t_{i}^{\left(0\right)}-\sum_{j\neq i}^{M}W_{i}{\left(H_{i,i}W_{i}\right)}^{-1}H_{i,j}t_{j}^{\left(0\right)}.$$

The second term of Equation (5) is the interference signal replicas to cancel the second term of Equation (4) at the receiver side. From Equations (4) and (5), the new reception signal vector $r_{i}^{\left(1\right)}$ is rewritten as,

(6) $$\begin{array}{ccc} r_{i}^{\left(1\right)} & = & \sum_{j}^{M}H_{i,j}t_{j}^{\left(1\right)}+n_{i}\\ & = & H_{i,i}t_{i}^{\left(0\right)}+\sum_{j\neq i}^{M}H_{i,j}t_{j}^{\left(0\right)}\\ & & -\sum_{j\neq i}^{M}H_{i,j}t_{j}^{\left(0\right)}-\sum_{j\neq i}^{M}H_{i,j}\sum_{j\neq i}^{M}W_{j}{\left(H_{j,j}W_{j}\right)}^{-1}H_{j,k}t_{k}^{\left(0\right)}+n_{i}\\ & = & H_{i,i}t_{i}^{\left(0\right)}-\sum_{j\neq i}^{M}H_{i,j}\sum_{j\neq i}^{M}W_{j}{\left(H_{j,j}W_{j}\right)}^{-1}H_{j,k}t_{k}^{\left(0\right)}+n_{i}. \end{array}$$

The ICLI cancellation scheme replaces the second ICLI term of Equation (4) with that of Equation (6). This replaced term is defined as residual ICLI. If the power of residual ICLI is smaller than that of original one, the ICLI cancellation becomes effective. Here formulates its required condition. As stated, the intensity of the second term of Equation (4) needs to be relatively smaller than that of the first term:

(7) $$∥H_{i,i}W_{i}s_{i}∥>∥\sum_{j\neq i}^{M}H_{i,j}W_{j}s_{j}∥,$$

where ∥.∥ denotes the Frobenius norm. By multiplying ${\left(H_{i,i}W_{i}\right)}^{-1}$, we can obtain

(8) $$∥s_{i}∥>∥\sum_{j\neq i}^{M}{\left(H_{i,i}W_{i}\right)}^{-1}H_{i,j}W_{j}s_{j}∥.$$

Multiplying transmission weight and summing up around *M* clusters except for the *i*-th one, the following relationship is obtained:

(9) $$∥\sum_{j\neq i}^{M}H_{i,j}t_{j}^{\left(0\right)}∥>∥\sum_{j\neq i}^{M}H_{i,j}\sum_{k\neq j}^{M}W_{j}{\left(H_{j,j}W_{j}\right)}^{-1}H_{j,k}t_{k}^{\left(0\right)}∥.$$

According to Equation (9), the intensity of the second term of Equation (6) should be smaller than that of Equation (4). It indicates that the ICLI can be suppressed when the condition in Equation (7) is satisfied. The first order residual ICLI in the second term of Equation (6) can be further suppressed via higher order cancellation, i.e., generating the ICLI signal replicas in an iterative manner. The transmission signal vector with the second order ICLI cancellation $t_{i}^{\left(2\right)}$ can be generated to replicate the residual ICLI term in Equation (6).

(10) $$\begin{array}{ccc} t_{i}^{\left(2\right)} & := & t_{i}^{\left(0\right)}-\sum_{j\neq i}^{M}W_{i}{\left(H_{i,i}W_{i}\right)}^{-1}H_{i,j}t_{j}^{\left(0\right)}\\ & & +\sum_{j\neq i}^{M}W_{i}{\left(H_{i,i}W_{i}\right)}^{-1}H_{i,j}\sum_{k\neq j}^{M}W_{j}{\left(H_{j,j}W_{j}\right)}^{-1}H_{j,k}t_{k}^{\left(0\right)}\\ & = & t_{i}^{\left(0\right)}-\sum_{j\neq i}^{M}W_{i}{\left(H_{i,i}W_{i}\right)}^{-1}H_{i,j}\left\{t_{j}^{\left(0\right)}-\sum_{k\neq j}^{M}W_{j}{\left(H_{j,j}W_{j}\right)}^{-1}H_{j,k}t_{k}^{\left(0\right)}\right\}. \end{array}$$

We can see the expression of $t_{i}^{\left(2\right)}$ in Equation (10) contains the identical expression of $t_{i}^{\left(1\right)}$ in Equation (5); hence, Equation (10) can be simplified as,

(11) $$t_{i}^{\left(2\right)}=t_{i}^{\left(0\right)}-\sum_{j\neq i}^{M}W_{i}{\left(H_{i,i}W_{i}\right)}^{-1}H_{i,j}t_{j}^{\left(1\right)}.$$

The α-th and (α−1)-th order transmission signal vectors are related to each other. Finally, general expression of higher order ICLI cancellation can be derived as,

(12) $$\begin{array}{ccc} t_{i}^{\left(\alpha \right)} & = & t_{i}^{\left(0\right)}-\sum_{j\neq i}^{M}G_{i,j}t_{j}^{\left(\alpha -1\right)}, \end{array}$$

(13) $$\begin{array}{ccc} G_{i,j} & = & W_{i}{\left(H_{i,i}W_{i}\right)}^{-1}H_{i,j}. \end{array}$$

Iteratively calculating the recurrence Equation (12) yields the higher order ICLI cancellation in a simple manner. The resulting received signal vector $r_{i}^{\left(\alpha \right)}$ is given by,

(14) $$\begin{array}{ccc} r_{i}^{\left(\alpha \right)} & = & \sum_{j}^{M}H_{i,j}t_{j}^{\left(\alpha \right)}+n_{i}\\ & = & H_{i,i}W_{i}s_{i}+{\left(-1\right)}^{\alpha }\sum_{j\neq i}^{M}H_{i,j}\sum_{k(1)\neq j}^{M}G_{j,k(1)}\cdots \sum_{l\neq k(\alpha )}^{M}G_{k(\alpha ),l}W_{l}s_{l}+n_{i}. \end{array}$$

When the condition in Equation (7) is well satisfied, the intensity of the second term of Equation (14) can be reduced as increasing the iteration order α.

Above feature of the ICLI cancellation scheme contributes to reduce complexity for cooperative transmission. It should be noted that Equation (12) contains only a single summation of the ICLI signals from interfering (M−1) clusters around the *i*-th cluster. This means that $t_{i}^{\left(\alpha \right)}$ can be calculated from the limited information of $G_{i,j}$ and $t_{j}^{\left(\alpha -1\right)}$, and their multiplication. Unlike the full cooperative MU-MIMO which necessitates the large size of CSI matrix operation, overall computation complexity can be lightened. It can be regarded that cooperation region is localized but continuously spread globally.

Here we assume Gram–Schmidt orthogonalization [20] as a MU-MIMO precoding algorithm. Although well-known precoding schemes such as zero-forcing (ZF) [21] or minimum mean square error (MMSE) [22] can be possible, such simpler schemes have property to normalize channel (propagation) gain. In multiuser environment, it indicates that throughput performance of users who locate far from BS would be improved, whereas users nearby BS cannot enjoy higher throughput. On the other hand, Gram–Schmidt orthogonalization can keep channel gain relationship among users while canceling interference components. In order not to break such unfairness depending on original user locations, we employed Gram–Schmidt orthogonalization as a basic precoding scheme.

### 2.2. Computation Complexity

The ICLI cancellation offers a reduced computation complexity. Defined as the number of multiplications, its quantitative values are disclosed. In the case of the full cooperative MU-MIMO based on Gram–Schmidt orthogonalization, the complexity required for CU can be estimated as;

(15) $$\Phi_{conv}=L^{3}\left(L+1\right)+L^{2}.$$

The above includes transmission weight matrix calculation (W←H) and multiplication with the transmission symbol (Ws). As described, full cooperation requires computation complexity with order of $O(L^{4})$ exponentially increased with the number of cooperating RUs. On the other hand, multiplications required for the ICLI cancellation is derived from Equations (12) and (13);

(16) $$\begin{array}{ccc} \Phi_{prop} & = & M\{\left(4+M\right)C^{3}+\left(1+\alpha M\right)C^{2}\}\\ & = & L(4C^{2}+LC+C+\alpha L). \end{array}$$

Complexity order of our approach is accordingly $O(\alpha L^{2}C^{2})$. When values of α and *C* are up to three, respectively, complexity only depends on $L^{2}$. It obviously contributes to significant complexity reduction.

**Figure 2:**
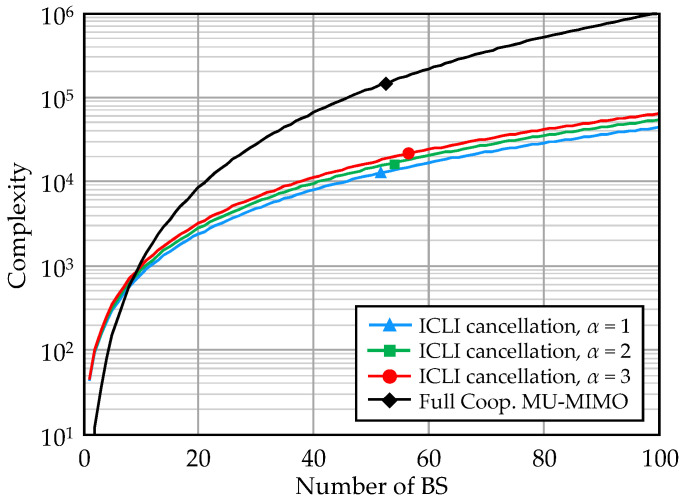
Computation complexity (C=3).

Figure 2 compares the number of multiplications required for the signal processing with the full cooperative MU-MIMO and the ICLI cancellation. The complexity of the clustering case is plotted for C=3. When L>10, the ICLI cancellation yields lower complexity than full cooperative MU-MIMO. It indicates that our scheme is applicable to even such small scale UHD-DAS cooperation. In addition, impact of increasing cancellation order α is slight, although the spectral efficiency improvement almost saturates at α=3.

## 3. Proposed Scheme: Dynamic Clustering and Coordinated User Scheduling

As formulated above, the requirement in Equation (7) should be satisfied to appropriately obtain the ICLI cancellation ability. Our previous study was based on strict frequency reuse or FFR to yield this condition. However, it lessens spectral efficiency due to the bandwidth division loss under the constraint of limited frequency resource. To leverage the interference cancellation approach with the single frequency network, here, dynamic clustering and coordinated user scheduling are introduced.

**Figure 3:**
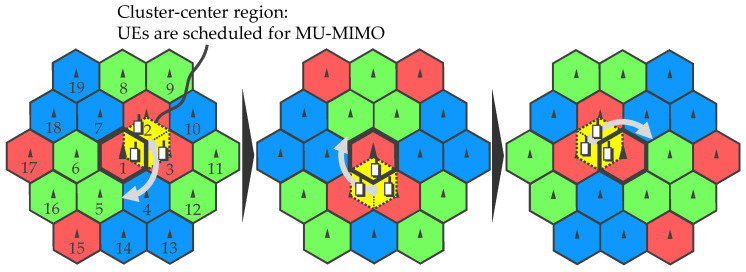
Dynamic clustering and scheduling region.

**Figure 4:**
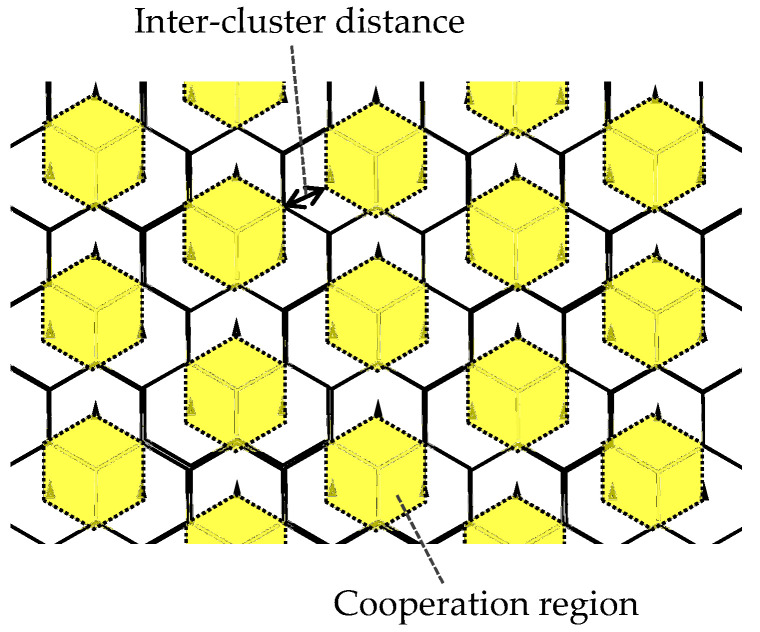
Inter-cluster interference provided by dynamic clustering and scheduling.

Figure 3 illustrates conceptual procedure of the proposed dynamic clustering. Suppose three RUs form one cooperating cluster, i.e, *C* = 3, and its clustering patterns are switched periodically. It can be rephrased as that each RU changes cooperating comrades with predetermined intervals. Let $z_{i}^{\beta }\in N^{1\times C}$ denote the set of cell (RU) indices forming the *i*-th cluster at the βth state (β=0,1,2), RUs for the 1st (center) cluster cyclically transitions as $z_{1}^{0}=\left\{1,2,3\right\}\to z_{1}^{1}=\left\{1,4,5\right\}\to z_{1}^{2}=\left\{1,6,7\right\}\to \ldots$. In this case, cooperation region is determined as intra-cluster region so as to avoid ICLI. Three UEs are selected from the region at each stage by observing instantaneous signal strength in a round robin manner. As a result, virtual cells can keep reuse distance as shown in Figure 4; the ICLI cancellation is expected to be effectively work. Then, cooperative transmission via MU-MIMO is performed to the cluster basis and then the ICLI cancellation is additionally applied.

## 4. Computer Simulation

### 4.1. Simulation Parameters

System level simulation parameters are listed in Table 1. Clusters are hexagonally sited with the single frequency reuse and the characteristics of the center cluster are the focus. In the evaluation, *M* clusters are taken into account in the area where interference-to-noise power ratio (INR) observed at the center cluster is more than −20 dB. $\tilde{K}=50$ UEs are uniformly distributed in each hexagonal cell and scheduled for downlink transmission with observing their signal strength in descending order. MU-MIMO based on Gram–Schmidt orthogonalization [20] is applied to the cluster basis. Transmission power for each RU is determined by average reception SNR for UEs locating in the cell edge; here, assume that the cell edge SNR = 20 dB. The total transmission power of all RUs is assumed to be constant in order to compare each method appropriately in terms of reception signal-to-interference and noise power ratio (SINR). Evaluation metric is achievable spectral efficiencies for several parameters of ICLI cancellation order α.

The spectral efficiency of the *u*-th UE in the *i*-th cluster achieved by the α-th order ICLI cancellation on the downlink is defined as,

(17) $$\Gamma_{u,prop}=\log_{2}\left(1+\frac{\left(h_{i,u}w_{i,u}\right){\left(h_{i,u}w_{i,u}\right)}^{H}}{\sum_{k\neq u}^{C}\left(h_{i,u}w_{i,k}\right){\left(h_{i,u}w_{i,k}\right)}^{H}+q_{i,u}q_{i,u}^{H}+\sigma_{n}^{2}}\right),$$

where

(18) $${\left(q_{i,1},\cdots ,q_{i,C}\right)}^{T}=\sum_{j\neq i}^{M}H_{i,j}\sum_{k(1)\neq j}^{M}G_{j,k(1)}\cdots \sum_{l\neq k(\alpha )}^{M}G_{k(\alpha ),l}W_{l}.$$

${\left(.\right)}^{H}$ denotes the Hermitian transpose. $h_{i,u}\in C^{1\times C}$ is the *u*-th row vector of $H_{i,i}$, $w_{i,u}\in C^{C\times 1}$ is the *u*-th column vector of $W_{i}$, and the *u*-th row vector $q_{i,u}\in C^{1\times C}$ indicates a suppressed residual ICLI derived from the second term of Equation (14). $\sigma_{n}^{2}$ is the receiver noise variance. $n_{u}$ is the *u*-th noise element of $n_{i}$. Here, assume perfect CSI estimation, i.e., ICLI term $\sum_{k\neq u}^{C}\left(h_{i,u}w_{i,k}\right){\left(h_{i,u}w_{i,k}\right)}^{H}=0$. Comparison scheme is the full cooperative MU-MIMO transmission. Its spectral efficiency of the *u*-th UE can be represented as,

(19) $$\Gamma_{u,conv}=\log_{2}\left(1+\frac{\left(h_{u}w_{u}\right){\left(h_{u}w_{u}\right)}^{H}}{\sum_{k\neq u}^{L}\left(h_{u}w_{k}\right){\left(h_{u}w_{k}\right)}^{H}+\sigma_{n}^{2}}\right),$$

where $h_{u}\in C^{1\times L}$ and $w_{u}\in C^{L\times 1}$ denote the *u*-th row vector of whole CSI matrix H and the *u*-th column vector of $W\in C^{L\times K}$, respectively. Here, assume a narrow-band flat fading channel condition and the proposed dynamic clustering and coordinated scheduling are applied to the subcarrier basis. It can be regarded as specified one subcarrier of OFDM. Detailed system level simulation process is summarized in Algorithm 1; it is repeated with different channel origination to obtain sufficient samples of spectral efficiency values. With the full cooperative MU-MIMO case as a comparison scheme, Steps 12–15 are alternated to W←Gram_Schmidt(H) and spectral efficiency calculation using Equation (19).

**Algorithm 1** Simulation process.
**Initialization:**
1:Set the scheduled UEs per cluster: $\phi_{i}=\emptyset$, i=1,…,M2:Set the UEs per cell: $X\left(j\right)\in \left\{1,\ldots ,\tilde{K}\right\}$3:Set the cells (RUs): Y∈{1,…,j,…,L}4:Set the clusters at the βth clustering state: $Z_{i}\in \left\{\left\{z_{1}^{\beta }\right\},\ldots ,\left\{z_{M}^{\beta }\right\}\right\}$, β=0,1,2
**Procedure:**
5:
n←0
6:
**while**
|X(j)|>0
**do**
7:  Determine cluster sets: β←nmod38:  **for**
i=1 to *M*
**do**9:    $\phi_{i}\leftarrow Coordinated\_Scheduling\left(z_{i}^{\beta }\right)$10:    $X\left(z_{i}^{\beta }\right)\leftarrow X\left(z_{i}^{\beta }\right)\backslash \phi_{i}$11:  **end for**12:  Construct channel matrix: $H=\{H_{i,i}\}$13:  $W_{i}\leftarrow Gram\_Schmidt\left(H_{i,i}\right)$14:  Calculate residual interference term in Equation (18)15:  Calculate spectral efficiency in Equation (17)16:  n←n+117:
**end while**
18:**function**Coordinated_Scheduling($z_{i}^{\beta }$)19:  Find UEs $x\in X(z_{i}^{\beta })$ locating in intra-cluster region20:  **return**
*x*21:
**end function**


### 4.2. Simulation Results: Clustering and Scheduling Effect

**Figure 5:**
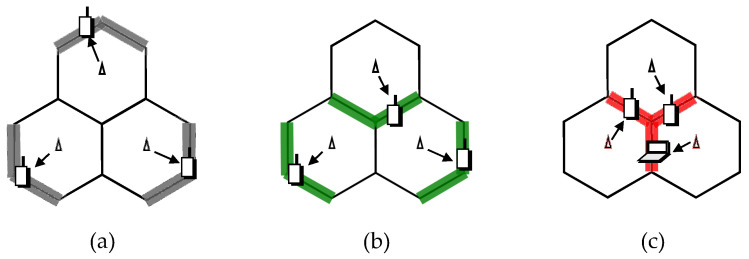
Coordinated UE scheduling strategies: (**a**) Case 1, all UEs locate in cluster-edge region; (**b**) Case 2, two UEs locate in cluster-edge and 1 UE is in center region; and (**c**) Case 3, all UEs locate in cluster-center region.

First, the coordinated scheduling effect is examined as a preliminary study. Figure 5 shows user scheduling strategies exemplified as

- Case 1: All three UEs locate at cluster-edge region.
- Case 2: Two UEs locate at cluster edge and 1 UE at cluster center regions.
- Case 3: All three UEs locate at cluster-center region.

UEs are uniformly distributed one by one in each colored region, respectively. The above cases are compared in terms of channel capacity normalized per cell. It is defined as,

(20) $$\gamma =\log_{2}\left[\det\left\{I+\frac{1}{C\sigma_{n}^{2}}\left(H_{i,i}W_{i}\right){\left(H_{i,i}W_{i}\right)}^{H}\right\}\right]$$

**Figure 6:**
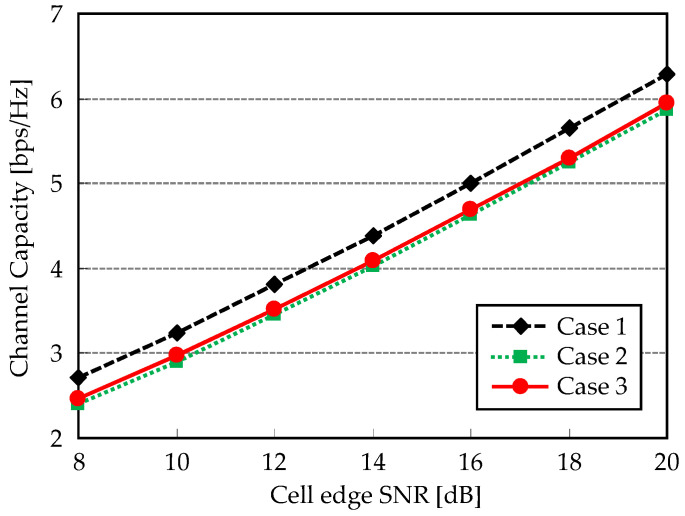
Channel capacity versus cell-edge SNR.

Figure 6 shows channel capacities for three cases. It equivalently represents the spectral efficiency without ICLI impact. Case 1 exhibits the largest channel capacity since location of UEs provides lower spatial correlation. It is preferred to obtain the spatial multiplexing gain. Case 3 shows the second best performance. Although the spatial correlation becomes large, which causes orthogonalization loss of MU-MIMO transmission, diversity gain can be obtained thanks to shortened distance to RUs. As for Case 2, neither multiplexing gain nor diversity gain can be produced, and thus such irregular condition shows the lowest channel capacity.

**Figure 7:**
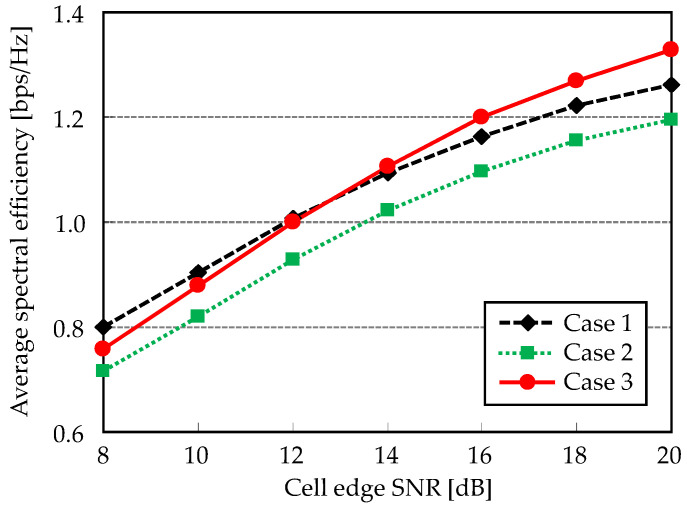
Spectral efficiency versus cell-edge SNR.

The next evaluation considers ICLI impact. Spectral efficiency performances for three cases are plotted in Figure 7. Here, ICLI cancellation is not applied, i.e., α=0. When cell edge SNR is low, superiority for each case follows the above channel capacity characteristics. It indicates that the impact of orthogonalization loss in Case 3 is larger than ICLI effect in Case 1. As the cell edge SNR increases, ICLI becomes significant and thus interference avoidance effect provided by the Case 3 outperforms other cases. It can be observed that cross point is around 13 dB. Spectral efficiency of Case 2 is also the lowest. Inferior channel capacity is further affected by ICLI. As a result, coordinated scheduling such that UEs locate in intra-cluster region could be effective under the ICLI existence. Therefore, our ICLI cancellation will also effectively work in such situation.

### 4.3. Simulation Results: Dynamic Clustering and Inter-Cluster Interference Cancellation

**Figure 8:**
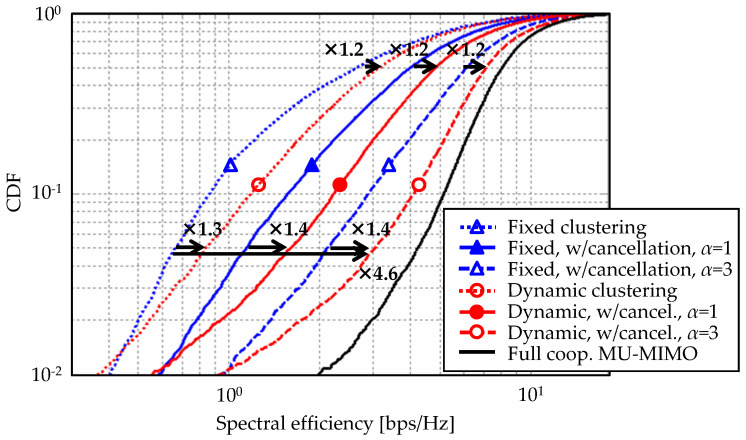
CDFs of spectral efficiency.

Finally, spectral efficiency improvement by the overall proposed approach is assessed via large scale UHD-DAS. Figure 8 plots cumulative distribution functions (CDFs) of the spectral efficiency upon the application of dynamic clustering and ICLI cancellation. Setting baseline as fixed clustering, introducing dynamic clustering provides 30–40% gain at CDF = 5% region, which mainly represents cell-edge characteristics. As for the median values, 20% improvement is also confirmed. It can successfully leverage effectiveness of ICLI cancellation. Increasing ICLI cancellation order to the third (α=3), its spectral efficiency improvement at cell-edge region is 360% and resultant overall CDF characteristics approximately approaches that of full cooperative MU-MIMO. As described in Section 2.1, the ICLI cancellation scheme subtracts interference replica signals before transmission. Although it contributes to cancel major ICLI components causing performance degradation, it leaves minute ICLI, i.e., residual interference, as shown in the second term of Equation (14). In the evaluation condition in this paper, α=3 provides the optimal condition where ICLI power is minimized. Since the power of interference to be canceled is initially small thanks to dynamic clustering, a few iterative cancellations is enough. Our past evaluation also clarified that up to three iterations can exhibit almost saturated interference cancellation performance [16].

As expected, the dynamic clustering virtually constructs coordinated cell in intra-cluster region which can equivalently realize frequency reuse condition. The proposed approach also offers a reduced computation complexity for preprocessing, whereas it can achieve high spectral efficiency improvement without any channel bandwidth division loss. Although simulative evaluation assumed a narrow-band flat fading channel condition to which the proposed scheme are applied, it can be applied for grouped subcarriers, i.e., subbands. Its operation granularity can be arbitrary chosen depending on capability of CU or required communication quality. From the above results, it can be a promising technique for efficient small cell deployment with cooperative transmission.

## 5. Conclusions

This paper proposes dynamic clustering and coordinated scheduling on the inter-cluster interference cancellation for ultra-high density distributed antenna systems. The proposed approach enables our previous interference cancellation scheme to effectively work even in a single frequency reuse condition. Computer simulation valuably elucidated its spectral efficiency performance, which approximately approaches that for full cooperative multiuser MIMO transmission. It can be concluded that the overall proposal is one of the most promising solutions to implement spectrally efficient large scale cooperation.

## Figures and Tables

**Table 1:** Simulation parameters.

Parameters	Values
Cell deployment	Hexagonal
Number of UE	50 per cell
Cell edge SNR	20 dB
Cluster Size *C*	3
Reuse Factor	1
Inter-cluster interference cancellation order α	1, 3
MU-MIMO transmission weight	Gram–Schmidt orthogonalization [20]
Carrier frequency	2 GHz
Propagation model	ITU-R M.1225 Pedestrian B [23] 40.1log${}_{10}$ (*d* [m])+39 dB
Fading model	i.i.d Rayleigh
RU/UE antenna	Single, Omni directional

## Abbreviations

The following abbreviations are used in this manuscript:

UHD-DAS	Ultra-High Density Distributed Antenna System
MIMO	Multiple-Input Multiple-Output
MU-MIMO	Multiuser MIMO
Wi-Fi	Wireless Fidelity
WiMAX	Worldwide Interoperability for Microwave Access
LTE	Long Term Evolution
BS	Base Station
UE	User Equipment
CoMP	Coordinated MultiPoint
CU	Central Unit
RU	Radio Unit
ICI	Inter-Cell Interference
ICLI	Inter-Cluster Interference
CSI	Channel State Information
FFR	Fractional Frequency Reuse
AWGN	Additive White Gaussian Noise
SNR	Signal-to-Noise power Ratio
SINR	Signal-to-Interference and Noise power Ratio
CDF	Cumulative Distribution Function
